# Abundant resources compensate for the uneven distribution of ungulates in desert grassland

**DOI:** 10.3389/fpls.2024.1421998

**Published:** 2024-07-26

**Authors:** Xiaowei Gou, Atsushi Tsunekawa, Mitsuru Tsubo, Fei Peng, Yunxiang Cheng

**Affiliations:** ^1^ Department of Grassland Resource and Ecology, College of Grassland Science and Technology, China Agricultural University, Beijing, China; ^2^ International Platform for Dryland Research and Education, Tottori University, Tottori, Japan; ^3^ Arid Land Research Center, Tottori University, Tottori, Japan; ^4^ Key Laboratory of Desert and Desertification, Northwest Institute of Eco-Environment and Resources, Chinese Academy of Sciences, Lanzhou, China; ^5^ Department of Ecology and Environment, Inner Mongolia University, Hohhot, China

**Keywords:** foraging distribution, overgrazing, resource selection function model, management strategies, resource availability, desertification

## Abstract

**Introduction:**

Strategically managing livestock grazing in arid regions optimizes land use and reduces the damage caused by overgrazing. Controlled grazing preserves the grassland ecosystem and fosters sustainability despite resource limitations. However, uneven resource distribution can lead to diverse grazing patterns and land degradation, particularly in undulating terrains.

**Methods:**

In this study, we developed a herbivore foraging algorithm based on a resource selection function model to analyze foraging distribution patterns, predict the probability of foraging, and identify the determinants of foraging probability in cattle. The study area was a complex desert landscape encompassing dunes and interdunes. Data on cattle movements and resource distribution were collected and analyzed to model and predict foraging behavior.

**Results:**

Our findings revealed that cattle prefer areas with abundant vegetation in proximity to water sources and avoid higher elevations. However, abundant resource availability mitigated these impacts and enhanced the role of water points, particularly during late grazing periods. The analysis showed that available resources primarily determine foraging distribution patterns and lessen the effects of landforms and water distance on patch foraging.

**Discussion:**

The results suggest that thoughtful water source placement and the subdivision of pastures into areas with varied terrain are crucial for sustainable grazing management. By strategically managing these factors, land degradation can be minimized, and the ecological balance of grassland ecosystems can be maintained. Further research is needed to refine the model and explore its applicability in other arid regions.

## 1 Introduction

Herbivore grazing is a widespread land-use practice that provides various benefits to society, such as food and income (Kang and Akinnifesi, 2000; Fuhlendorf and Engle, 2001; Teague and Kreuter, 2020). However, with the projected increase in the population and the corresponding demand for meat, the total number of livestock is expected to double by 2050, leading to overgrazing problems in grasslands (Rosegrant et al., 2009). Overgrazing can alter ecosystem functions, reduce vegetation nutrients and productivity, and ultimately lead to grassland degradation, especially in arid and semi-arid regions globally (Ayantunde et al., 1999; Gutman et al., 1999; Bardgett et al., 2021). In addition to the increasing number of livestock per unit area, poor livestock grazing distribution can contribute to overgrazing, since livestock tend to overuse preferred areas, leaving other areas only lightly grazed (Launchbaugh and Howery, 2005; Dwyer et al., 2021). Therefore, understanding the distribution of livestock grazing is critical for preventing grassland degradation and aiding restoration through grazing management (DeYoung et al., 2000; Briske et al., 2008; Hao et al., 2018).

The spatial distribution of livestock grazing is largely determined by the theory of maximum energy intake, which posits that livestock aim to maximize their energy intake while minimizing energy costs (Bailey, 2005). Abiotic and biotic factors, such as the location of water points, landform characteristics, and pasture quality and quantity, affect the trade-offs between energy intake and cost, and thus, define the spatial distribution of grazing (Pyke, 1984). In relatively flat areas, livestock tend to concentrate around water points as they require regular access to water, thereby conserving energy that would be expended searching for foraging opportunities. However, consecutive grazing around water points can lead to a decrease in forage availability, ultimately forcing livestock to expand to further areas in search of high-quality and -quantity herbage (Scasta et al., 2016). As livestock move further away from water points, net energy intake decreases because of the additional energy expended while traveling back and forth between the water point and newly found patches (Al-Khaza’leh et al., 2015). Consequently, the grazing pressure shifts temporally from areas near water points during the early grazing period to patches farther away at the end of the grazing period (Ludwig et al., 1999). Thus, we hypothesized that resource availability and distance to a water point directly affects foraging probability, even in pastures with rugged terrain.

The rugged topography of grazing lands creates a heterogeneous arrangement of environmental factors that can lead to distinct spatial distribution patterns for livestock. The location of water points and variations in elevation are significant in this context, as it influences the movement patterns of livestock (Piechnik et al., 2012; Bailey et al., 2015). Livestock may move to a relatively high-altitude area to maximize net energy intake rather than walking long distances to find new patches (Piechnik et al., 2012). However, rugged terrain can also lead to an uneven grazing distribution, with livestock spending more time on gentle terrain, leaving other areas ungrazed (Mueggler, 1965; Ganskopp and Vavra, 1987; Bailey et al., 2015). For example, cattle avoid foraging areas with slopes with an incline > 20% (Gillen et al., 1984). The movement patterns of livestock and the consequent grazing pressure distribution become more complicated in rugged topography, affecting the forage availability and net energy intake due to the physiological requirements of climbing and loading on rugged landforms (Brandyberry et al., 1991).

The direct effect of rugged topography on energy expenditure during livestock foraging is compounded by its indirect effect on plant community composition, which varies with altitude (Miyasaka et al., 2011). As resource availability declines on a ranch, animals move to higher elevations, where resource availability is more abundant (Wilmshurst et al., 1999). However, the trade-off between the distance to water points and the climb to higher elevations for selective foraging remains unclear. Thus, we hypothesized that elevation in rugged landforms directly and indirectly impacts the probability of foraging via their interaction with resource availability and distance to water points, respectively. In rugged areas, patches selected for grazing will experience a higher grazing pressure compared to those in flatter areas, making them more prone to degradation (Bailey, 2005).

The Horqin Sandy Land in northern China has experienced severe desertification (Zuo et al., 2008). Despite efforts to restore degraded grasslands by reducing livestock numbers and grazing time through the establishment of fences, desertification continues to be a challenge in this area (Miao et al., 2015). The rugged microtopography of landforms in this region creates a complex interplay between livestock distribution and landforms as well as the relative conditions of herbage. While previous studies have used the number of livestock per unit area to estimate grazing density and its impact on plant communities and soil properties (Li et al., 2012; Tang et al., 2016; Zhang et al., 2017), they have often neglected the spatiotemporal dynamics of actual foraging pressures. Furthermore, few studies have quantified the spatial distribution of cattle foraging on fine-scale ranches (approximately 20 ha). Our previous study revealed that cattle spent more time foraging in lowland than in dune areas as herbage conditions declined during the growing season (Gou et al., 2020). However, with only one season of data, it is difficult to capture the nature of the grazing distribution and its determinants. Thus, in this study, we used the resource selection function (RSF) to analyze foraging location data, predict the probability of foraging, and identify the determinants of foraging probability in cattle.

## 2 Materials and methods

### 2.1 Study area

The study was conducted on a family ranch located in the Horqin Sandy Land (42°00′N, 119°39′E) in northern China. The area is dominated by dunes (70% of the total area) with an average height, length, and width of 5–8 m, 400–600 m, and 20–40 m, respectively. The remaining 30% of the area is lowland (**Supplementary Figure S2**). The region experiences precipitation mainly between June and August, which accounts for 70–80% of the annual total, and the annual mean temperature was 7.3°C from 1980 to 2014. Sandstorms caused by strong winds are common during spring, with average wind speeds ranging from 3.2–4.5 m s^-1^ between March and May.

The dominant grazing livestock in Horqin Sandy Land are sheep, goats, and cattle. The study ranch has implemented the ‘Livestock-forage balance management’ policy (Yanbo et al., 2014), which allows field grazing only from 1st July to 30th September to reduce grazing pressures. Since the implementation of this policy, the average grazing density on the Horqin Sandy Land area has declined from 1.81 before 2010 to 0.19 sheep units.

The study ranch covers an area of 20.1 ha, with 8.04 ha of lowland and 12.06 ha of dunes. The vegetation is dominated by grass species, such as *Pennisetum centrasiaticum* and *Cleistogenes squarrosa*, and some dwarf shrubs, such as *Artemisia oxycephala* and *Artemisia halodendron*. The soil in the lowland areas is *Kastanozems*, whereas the dunes are comprised of Ustic *Sandic Entisols*, which are susceptible to wind erosion. The soil in the lowland areas contained more nutrients and higher soil moisture than the soil in the dunes. The rugged landform around the studied ranch represents a typical landform of the Horqin Sandy Land. The northwest of the ranch was used to grow corn and millet before the 1990s.

### 2.2 Herbage production and quality measurements

We selected a combination of seven uniformly spaced lowland locations and three characteristic dunes on the ranch to assess plant communities and biomass. Given the slight differences in species composition found in lowland regions, we chose a mix of three smaller and four larger lowland areas for a detailed examination of the herbaceous community. The dunes, predominantly situated along the ranch’s fence lines, included three centrally placed dunes for our analysis of dune vegetation. Data was collected on 15 July, August, and September in 2018 at each lowland site, where we randomly placed three quadrats (each 1 × 1 m) along a 10 × 10 m diagonal plot line. For the dunes, we positioned three additional quadrats: one on top of a dune, and one each on the leeward and windward slopes, making up 21 lowland and nine dune quadrats monthly.

We identified and recorded every plant species within these quadrats, harvested the aboveground plant parts, and stored these samples separately in envelopes. These samples were then dried at 55°C for 48 h before weighing to determine each species’ biomass. We calculated the total biomass per quadrat as the cumulative biomass of all plants within it. Samples from identical species across various lowland and dune quadrats were combined. Monthly, we assessed the crude protein (CP), neutral detergent fiber (NDF), acid detergent fiber (ADF), and total digestible nutrients (TDN) for each species, utilizing chemical analysis by Cumberland Valley Analytical Services in Tongzhou District, Beijing, China. The overall CP, NDF, ADF, and TDN for each quadrat were calculated as the average of these components in each species, adjusted for species’ relative abundance in the quadrat.

### 2.3 Grazing monitoring and data selection

In this study, we tracked the location of cattle using GPS devices (precision ± 3 m; catalog no. GT-600, i-gotU, Mobile Action Technology, Taipei).The total number of cattle on the ranch was thirteen, and all of them were initially fitted with GPS devices; however, owing to device malfunction, GPS data for two cattle were only available for specific periods in September. Therefore, GPS data for two adult Simmental cattle with 50 s recording intervals were used to represent the grazing pattern of the entire herd and the proportional grazing pressure at the ranch. The distance metrics (linear and cumulative distance) and turning angle were calculated using GPS records with time intervals ranging from to 100–800 s to relate instantaneous locations to short-period behavior. For two consecutive days (23 and 24 September, 2018; 09:00 to 17:00 UTC +8), we continuously recorded direct visual behavioral observations of one cattle, identifying that approximately 80% of its activities were grazing.

We used a Random Forest algorithm for categorizing various behaviors of livestock, utilizing both training metrics and behavioral data obtained from field observations. The efficacy of the Random Forest model was assessed by a 10-fold cross-validation approach, which segmented the data into distinct training and test subsets. This model demonstrated an overall accuracy rate of 87%, with a 95% confidence interval ranging between 85% and 90%. This allowed us to effectively categorize cattle positions into two primary behaviors: foraging and non-foraging (Gou et al., 2019).

### 2.4 Resource selection function and the foraging probability

To investigate the cattle’s preferred foraging areas, we examined several environmental variables, including slope, elevation, aspect, distance to water point (DWP), and the Normalized Difference Vegetation Index (NDVI) (**Supplementary Figure S3**). The digital surface models (DSM) map with a ground resolution of 2 × 2 m was generated according to the method described by Gou et al. (2020). Elevation was estimated from the DSM map, and slope (in degrees) and direction (north or south facing) were derived from the elevation layer. NDVI values were extracted from WorldView-2 satellite images with a 2-meter resolution on 16 July and 9 September to estimate the available vegetation resources. We assessed the effect of accessibility to water resources on cattle behavior by evaluating the Euclidean distance from the water point to each foraging pixel (2 × 2 m grid cell) on the ranch based on a DSM map using ArcGIS 10 (ESRI 2012). We also evaluated the interactive effects of the NDVI and elevation and distance from the water point on the probability of grid foraging.

To analyze the probability of an area being foraged by cattle at different times during the grazing season, we used the resource selection function (RSF), which is widely used to explore animal resource selection preferences (Boyce et al., 2002). The RSF was performed using a logistic regression model for the used (foraged points) and randomly generated available locations (Gillies et al., 2006; Manly et al., 2007). Both location types were annotated with each of the spatial variables mentioned above. We used the beta coefficients from each logistic regression model to estimate the RSF using the following equation:


$$w\left(x_{i}\right)=\frac{\exp\left(\beta_{1}x_{1i}+\ldots +\beta_{n}x_{ni}\right)}{1+\text{exp}\left(\beta_{1}x_{1i}+\ldots +\beta_{n}x_{ni}\right)}$$

where, $w\left(x_{i}\right)$ is the RSF and $\beta_{n}$ is the coefficient for the n-th predictor variable $x_{n}$ (Gillies et al., 2006; Manly et al., 2007).

**Figure 1:**
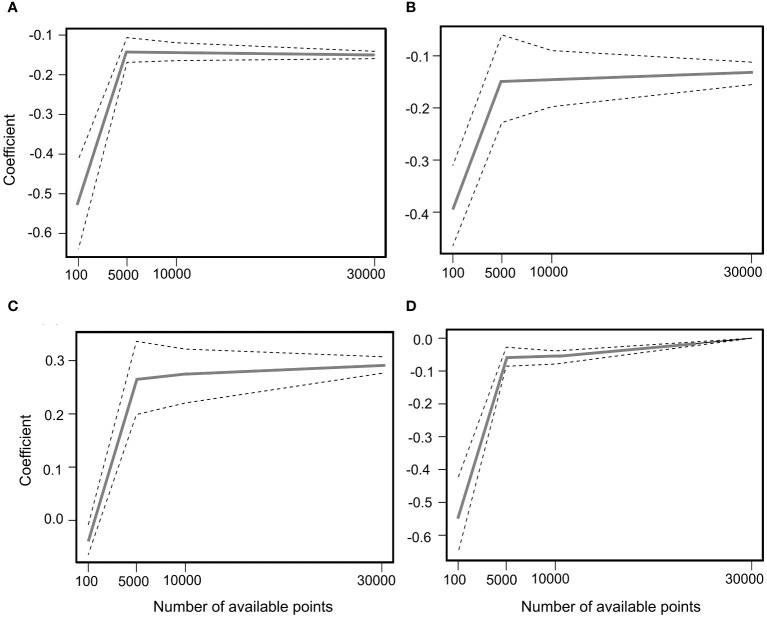
Coefficient estimates and 95% simulation envelopes (solid lines) from 500 RSF model iterations fitted to data simulated from variables: **(A)** Distance to Water Points (DWP), **(B)** Elevation, **(C)** NDVI, and **(D)** Slope during the July period.

**Figure 2:**
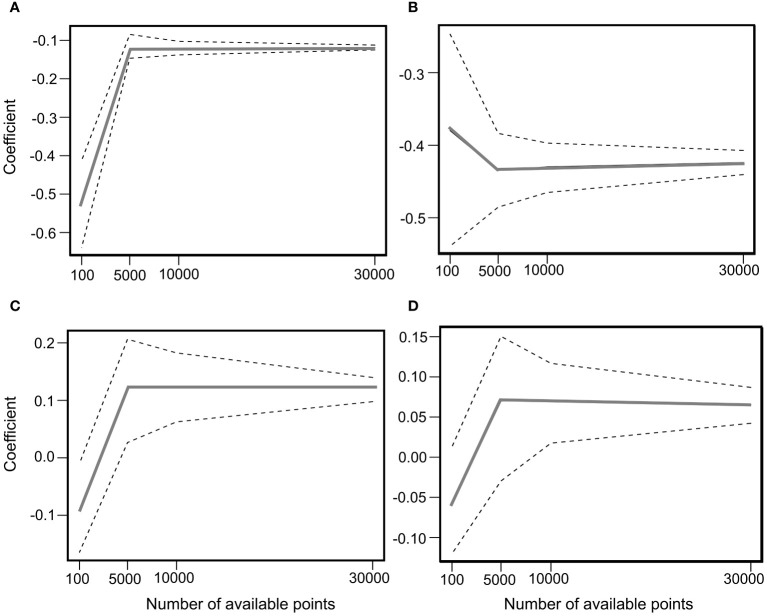
Coefficient estimates and 95% simulation envelopes (solid lines) from 500 RSF model iterations fitted to data simulated from variables: **(A)** Distance to Water Points (DWP), **(B)** Elevation, **(C)** NDVI, and **(D)** Slope during the September period.

Cattle-used (foraged, foraging behaviors) locations were generated from GPS records at 50 s time intervals of the classified foraging behaviors. To obtain an unbiased estimator of β with an adequate number of available location (Northrup et al., 2013; Roever et al., 2015), we increased the samples of random locations from the larger availability samples within the home range (ranch size, 100,000 grid cells) from 100, 1000, 5000, and 10000 to 30000 to fit β coefficients of logistic regression models. We repeated this process 1000 times and monitored the β coefficients of four representative covariates to identify the density at which coefficient values begin to converge. Convergence occurred at a minimum of 10,000 random locations in both the early and late RSF models (**Figures 1**, **2**). Therefore, we generated 100,000 available locations to construct early and late RSF models based on a logistic regression model.

### 2.5 Statistical analyses

To determine the best model fit, we used the Akaike Information Criterion (AICc) in the R software package MuMin, considering various combinations of logistic regressions (Anderson and Burnham, 2002; Grueber et al., 2011). We followed Burnham and Anderson’s (2002) recommendation to select the model with an AICc< 2 as the best fit (**Supplementary Tables S1**, **S2**).

For evaluating the fit between the predicted probabilities and actual observations, we segmented the predictive map into 20 uniform intervals based on the RSF, each representing a distinct probability range (0–5%, 5–10%, 10–15%, and so on). The observational data for the relevant time frame were overlaid on this map, and we analyzed the distribution frequencies of observed cattle locations across these RSF categories. While investigating the indirect effects of NDVI, moderated by landform features on RSF values, we applied a bootstrapping analysis to dissect significant NDVI-elevation interactions. The extent of these indirect effects was determined using the Johnson–Neyman (JN) technique (Kowalski et al., 1994), which helped estimate significant regions of standard deviation for the NDVI. The bootstrapping method yielded 95% bias-corrected confidence intervals from 50,000 samples, focusing on the lowest or most negatively significant standard deviation (SD) NDVI values, rounded to the nearest 0.05 SD as determined by the JN technique. In cases where the NDVI value did not reach statistical significance (P > 0.05), it was decreased by 0.05 SD increments until a significant SD deviation was achieved. Upon reaching significance, the last modified value was maintained.

## 3 Results

The herbage quality of biomass, as well as the quantity of CP and TDN, was significantly higher during the early grazing period compared to the late grazing period. However, the analysis of NDF and ADF yielded more intricate results when comparing the early and late grazing periods (**Supplementary Figure S1**).

**Figure 3:**
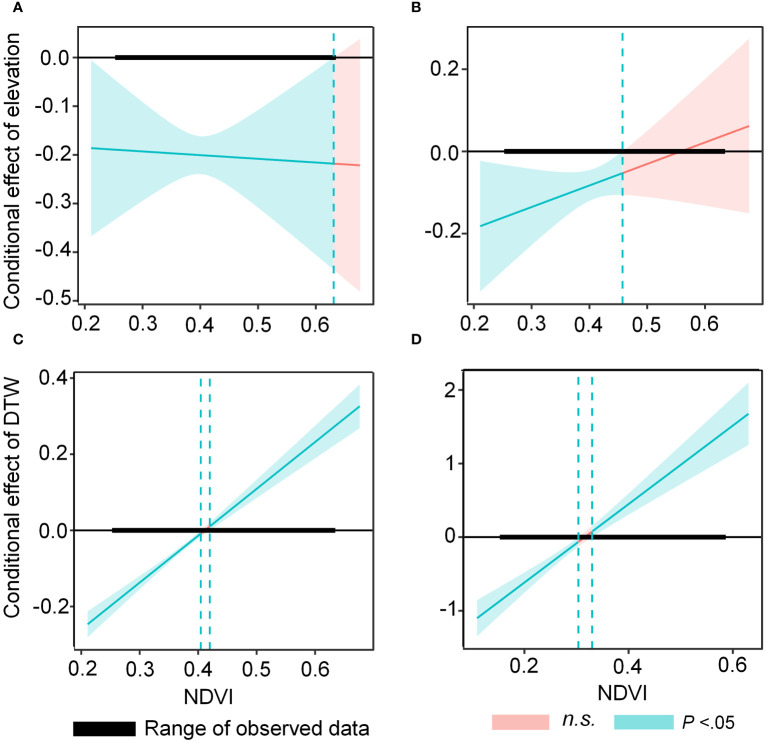
The conditional indirect effects of the Normalized Difference Vegetation Index (NDVI) on elevation during July **(A)** and September **(B)**, and on distance to water point (DWP) during July **(C)** and September **(D)**, as a function of RSF values. The Johnson–Neyman plot illustrates these effects with 95% confidence intervals.

The best supported model of cattle resource selection during the early grazing period (ΔAICc=0; **Supplementary Table S1**) included elevation, slope, the distance to water point (DWP), and the NDVI as explanatory variables. Models with AICc< 2 were also supported. During this period, the probability of cattle foraging selection was positively correlated with the NDVI but negatively correlated with the DWP, elevation and slope (**Table 1**). The beta (β) coefficients converg at -0.15 of DWP, -0.16 of elevation, 0.26 of NDVI, and -0.13 of Slope (**Figure 1**) The interaction between the NDVI, slope, and elevation was not significant; however, the interaction between the NDVI and DWP had a positive effect on cattle foraging selection. The negative and positive effects of DWP increased when the NDVI was< 0.4 or 0.43, respectively (**Figure 3**).

**Table 1:** Beta (β) coefficients of the standardized effects regression model explaining variations in habitat use by cattle in July and September.

Model	July	September
Predictor	β	β
Intercept	-0.67	-1.73
Elevation	-0.20*	-0.42*
Distance to water	-0.14*	-0.17*
Slope	-0.06	-0.06
NDVI	0.22*	0.20*
NDVI × Elevation	0.01	0.02*
NDVI × DWP	0.31*	0.49*

*indicates a significant relationship (P< 0.05).

The best model for explaining cattle resource selection during the late grazing period was the same as that during the early grazing period, including all variables (ΔAICc=0; **Supplementary Table S2**). Similar to the early grazing period, the NDVI and DWP had positive and negative effects, respectively, on the probability of cattle foraging selection. The beta (β) coefficients converg at -0.13 of DWP, -0.42 of elevation, 0.12 of NDVI, and 0.07 of Slope (**Figure 2**). Furthermore, the negative effect of elevation on resource selection was greater during the late grazing period than during the early grazing period (**Table 1**). The interactions between the NDVI, elevation, and the DWP were significantly positive during the late grazing period. The negative effects of elevation and DWP on resource selection decreased when the NDVI was< 0.44 and 0.3, respectively, while the positive effect of DWP increased when the NDVI was > 0.33 (**Figure 3**). The average NDVI decreased with increasing elevation during the early grazing period, whereas it increased slightly with increasing elevation during the late grazing period (**Figure 3**).

**Figure 4:**
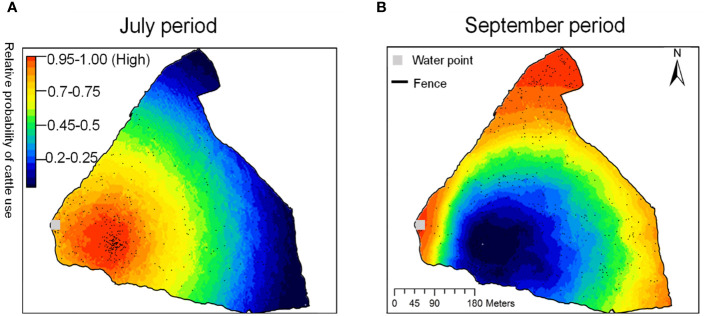
The predicted relative probability of cattle use in July **(A)** and September **(B)**. Areas of the highest relative probability of use are shown in red and areas of the lowest relative probability of use are shown in dark blue.

The results depict the cattle’s seasonal spatial behavior within our study area, applying the RSF across the entire area for both the beginning and end of the grazing season (**Figure 4**). During the initial grazing stage, we observed a pronounced concentration of cattle activity near the water source, while later in the season, their distribution expanded towards the ranch’s perimeter fences.

The accuracy of predictions was notably higher in the initial grazing phase compared to the latter. Specifically, in the early grazing phase, 72% of the cattle were found in areas exceeding 75% RSF, and 85% in areas above 50% RSF. By September, these figures shifted to 48% in areas over 75% RSF and 70% in areas above 50% RSF. In August, 30% of observed locations were in regions surpassing 75% RSF, while 60% were above 50% RSF, as presented in **Table 2**.

**Table 2:** Frequency distributions and percentage of cattle locations within equal RSF intervals during the early and late grazing periods.

RSF interval	Early grazing period		Late grazing period	
	Number of locations	Percentage locations	Number of locations	Percentage locations
0–5%	100	0.953%	251	2.41%
5–10%	169	1.611%	392	3.76%
10–15%	176	1.678%	302	2.90%
15–20%	157	1.497%	384	3.69%
20–25%	115	1.096%	302	2.90%
25–30%	167	1.592%	328	3.15%
30–35%	185	1.764%	390	3.74%
35–40%	199	1.897%	451	4.33%
40–45%	221	2.107%	384	3.69%
45–50%	247	2.355%	333	3.20%
50–55%	240	2.288%	301	2.89%
55–60%	193	1.840%	315	3.02%
60–65%	303	2.889%	311	2.99%
65–70%	285	2.717%	549	5.27%
70–75%	261	2.489%	463	4.45%
75–80%	344	3.280%	691	6.64%
80–85%	797	7.599%	794	7.62%
85–90%	777	7.408%	774	7.43%
90–95%	1039	9.907%	894	8.58%
95–100%	4513	43.030%	1805	17.33%
Total	10220		10414	

## 4 Discussion

### 4.1 Resource availability moderates the effects of the distance to water points on cattle foraging probability

Drinking water is essential for the survival and growth of livestock (Agouridis et al., 2005). It is difficult for livestock in arid and semi-arid regions to access adequate and reliable sources of drinking water (Usman et al., 2016). Thus, the strategic placement of water points has significant effects on the foraging behavior and distribution of grazing livestock. The livestock’s distance from the water source has been shown to influence the expansion of foraging selection, with livestock being concentrated in areas closer to water sources. As the distance to the water source increases, grazing intensity tends to decrease, suggesting that access to water is a crucial limiting factor for livestock distribution in arid and semi-arid regions (Holechek et al., 1989).

Therefore, the proximity and availability of water sources are critical factors that determine the spatial distribution and foraging behavior of livestock. This situation is only observed when resources are distributed evenly, as our findings (**Table 1**) and those of previous studies (Bailey et al., 1996; Ganskopp and Bohnert, 2009) suggest that resource availability affects the probability of foraging on a given patch. Specifically, increased resource availability enhanced the likelihood of foraging in a patch, ultimately affecting the spatial distribution of livestock grazing (**Table 1**; **Figures 3C, D**). Our findings demonstrate a significant interaction between the NDVI and DWP during the late grazing period (**Figure 3**). This suggests that cattle rely on multiple environmental cues to make decisions regarding their foraging locations based on prevailing conditions. Our results showed that when resources were abundant in the early grazing period, a slight increase in stress promoted grazing density through DWP (**Figure 3C**). The cattle were mainly concentrated at medium distance to the water point rather than staying very close or moving further from the water point to meet vegetation resource requirements (**Figure 4A**). This indicates that they had sufficient food options and did not need to return to water points to forage. In contrast, when resource availability was low, the effect of the DWP on cattle foraging probability was much stronger (**Figure 3D**). In this situation, cattle either stayed very close to water points to meet their water needs or moved farther away to satisfy their food requirements.

### 4.2 Resource availability moderates the effects of rugged landforms on cattle foraging probability

A previous study found that cattle tended to maximize their resource intake in lowlands and avoided higher dune elevations (Gou et al., 2020). However, the mechanisms through which resource availability moderates cattle selection in rugged landforms remains unclear. These findings are consistent with the fact that cattle seemed to avoid higher dune elevations during the early and late grazing periods. In addition, avoidance of dunes was stronger during the early compared with the late grazing period (**Figures 3A, B**; **Supplementary Figure S4**). This may be related to the energy costs of foraging, since the energetic costs of moving through rugged landforms are likely to affect the way animals navigate landscapes (Lundmark and Ball, 2008; Avgar et al., 2013). The energy costs of crossing rugged dunes varied between the beginning and end of the grazing period. In the early grazing period, cattle could easily fulfil their energetic and nutritional needs in lowland areas with less energy expenditure during traveling. Contrary to expectations, avoidance of dune elevation enhanced the net energy intake of foraging cattle and became less relevant as resource availability decreased during the late grazing period (**Supplementary Figure S1**). During the study period, cattle preferred higher areas with abundant vegetation resources and avoided sand dunes. This behavior can be attributed to the increased cost of traveling through dunes, which exponentially increases with a density below a certain threshold of support, leading to the avoidance of higher elevations (Parker et al., 1984). However, cattle may move to higher altitudes with sufficient herbage available to support foot loading. The results showed that the avoidance of foraging areas on dunes significantly decreased as vegetation resources increased to a threshold of 0.3 (**Figure 3B**). Such selection patterns may help offset energy deficits by minimizing the effort required to forage for ground herbage in sand dunes (Valdez and Krausman, 1999).

### 4.3 Implementations for livestock grazing management

Understanding the spatial requirements and resource requirements of livestock in response to increasingly variable and severe environmental conditions is crucial for meeting production goals and ensuring the sustainable conservation of pastures (Teague et al., 2013). Our study found that the distribution of cattle was influenced by seasonal changes in herbage conditions, the DWP, and the elevation of sand dunes. The variation in interactions led to an uneven distribution of cattle foraging, which was concentrated near water resources in the early grazing period and moved toward fence boundaries in the late grazing period.

Therefore, the strategic placement of supplements can be an effective tool for altering livestock distribution during the dry season (Bailey and Welling, 1999), which will likely be effective in attracting livestock to areas where grazing is desired and keeping livestock away from environmentally critical areas, such as riparian zones. This study showed that heavy foraging areas were located near water points in the early grazing period and near the fence boundary in the late grazing period; therefore, we would advise local farmers to encourage cattle to forage in areas far from the water point in the early grazing period and closer to it during the late grazing period.

However, supplemental sites are less attractive when green forage is abundant (Hightshoe et al., 1991). When a supplement was placed in rangeland pastures, the cattle congregated at the supplement site, grazed, and rested in adjacent areas within 600 m of the supplement site. Thus, although supplement placement strongly influences livestock distribution, it must be integrated with fencing, a water point, and other practices to achieve the grazing management goals.

## 5 Conclusion

This study enhances our understanding of how fluctuating herbage conditions influence cattle movements over various spatial and temporal scales. Leveraging granular environmental data and comprehensive cattle movement tracking, our model reveals patterns that can inform strategies to prevent excessive use of rangelands. In our analysis, we utilized resource function selection to assess the likelihood of cattle foraging in certain landforms, exploring various influencing factors. High grazing probabilities were linked to areas with increased NDVI values and proximity to water points, while higher elevations showed a lower grazing likelihood. Notably, as the grazing season transitioned from its early to late stages, the areas with higher grazing probabilities increasingly diverged from water points. During the early phase, grazing likelihood was inversely related to elevation yet positively correlated with NDVI. As the season progressed, the direct impacts of NDVI and elevation on grazing probabilities diminished, inducing the combined effect of NDVI and elevation. Thus, effective adaptive management of rangelands should account for the seasonal variability in cattle movement patterns, especially considering the dynamics of forage resource availability and its relationship with water proximity and dune elevation.

Furthermore, we suggest that local farmers consider seasonal management of grazing cattle to avoid ranch overgrazing. Creating grazing regimes in the early grazing period, where the core areas of cattle foraging are concentrated near the water point, separates the ranch into several groups to reduce foraging pressures and allows vegetation to regenerate more effectively. Additionally, forage supplements should be provided near the water point and lower dune areas to prevent cattle from walking longer distances on lowlands and moving upward into the dunes; thus, enabling them to save energy and maintain their body weight.

## Data availability statement

The raw data supporting the conclusions of this article will be made available by the authors, without undue reservation.

## Ethics statement

Ethical approval was not required for the studies involving animals in accordance with the local legislation and institutional requirements because this study did not involve direct interaction with live animals, nor did it include invasive procedures or manipulation of animal behavior. All data were collected through non-invasive observation and analysis of existing records, which does not fall under the purview of ethical review according to the guidelines of our institutional review board. Written informed consent was obtained from the owners for the participation of their animals in this study.

## Author contributions

XG: Conceptualization, Data curation, Formal analysis, Investigation, Methodology, Software, Writing – original draft, Writing – review & editing. AT: Project administration, Supervision, Writing – original draft, Writing – review & editing. MT: Resources, Visualization, Writing – original draft, Writing – review & editing, Supervision, Conceptualization. FP: Investigation, Methodology, Resources, Software, Supervision, Visualization, Writing – original draft, Writing – review & editing. YC: Conceptualization, Funding acquisition, Investigation, Resources, Writing – original draft, Writing – review & editing.
