# Supplementary material for: Abundant resources compensate for the uneven distribution of ungulates in desert grassland
Source: Front Plant Sci. 2024 Jul 26;15:1421998. doi: 10.3389/fpls.2024.1421998 (PMC11310146; doi:10.3389/fpls.2024.1421998)
Supplement: Supplementary file 1 [file DataSheet_1.docx]

Table S1 Model selection results for examining habitat use by cattle in July. The 5 highest-ranking models are presented.

| Model | Covariate composition | DF | AICc | ΔAICc | Weight |
| --- | --- | --- | --- | --- | --- |
| 1 | slope + elevation + aspect + dist to water + NDVI | 9 | 22813.2 | 48.9 | <0.001 |
| 2 | elevation + slope + dist to water + NDVI | 8 | 22764.3 | 0.0 | 0.975 |
| 3 | elevation + dist to water + NDVI | 8 | 22771.6 | 7.3 | 0.025 |
| 4 | slope+ dist to water + NDVI | 7 | Inf | Inf | <0.001 |
| 5 | slope + elevation + aspect + NDVI | 8 | 22822.9 | 58.6 | <0.001 |

Table S2 Model selection results for examining habitat use by cattle in September. The 5 highest-ranking models are presented.

| Model | Covariate composition | DF | AICc | ΔAICc | Weight |
| --- | --- | --- | --- | --- | --- |
| 1 | slope + elevation + aspect + dist to water + NDVI | 9 | 53697.2 | 53.1 | <0.001 |
| 2 | slope + elevation + dist to water + NDVI | 8 | 53644.1 | 0.0 | 1 |
| 3 | elevation + dist to water + NDVI | 8 | 53660.7 | 16.6 | <0.001 |
| 4 | slope + dist to water + NDVI | 7 | Inf | Inf | <0.001 |
| 5 | slope + elevation + aspect + NDVI | 8 | 53680.8 | 36.7 | <0.001 |

Fig S1 Comparison of herbage quality and quantity between the July and September grazing period. The box plot shows the values of (a) pasture ground biomass, (b) crude protein (CP), (c) total digestible nutrients (TDN), (d) neutral detergent fibre (NDF), (e) acid detergent fibre (ADF). In the box plot, the bounds of the box span from 25 to 75% percentile, the centre line represents the mean, and whiskers visualize 5 and 95% of the data points. (*P < .01, **P <.001).

Fig S2. Digital surface model (DSM) map of the study ranch


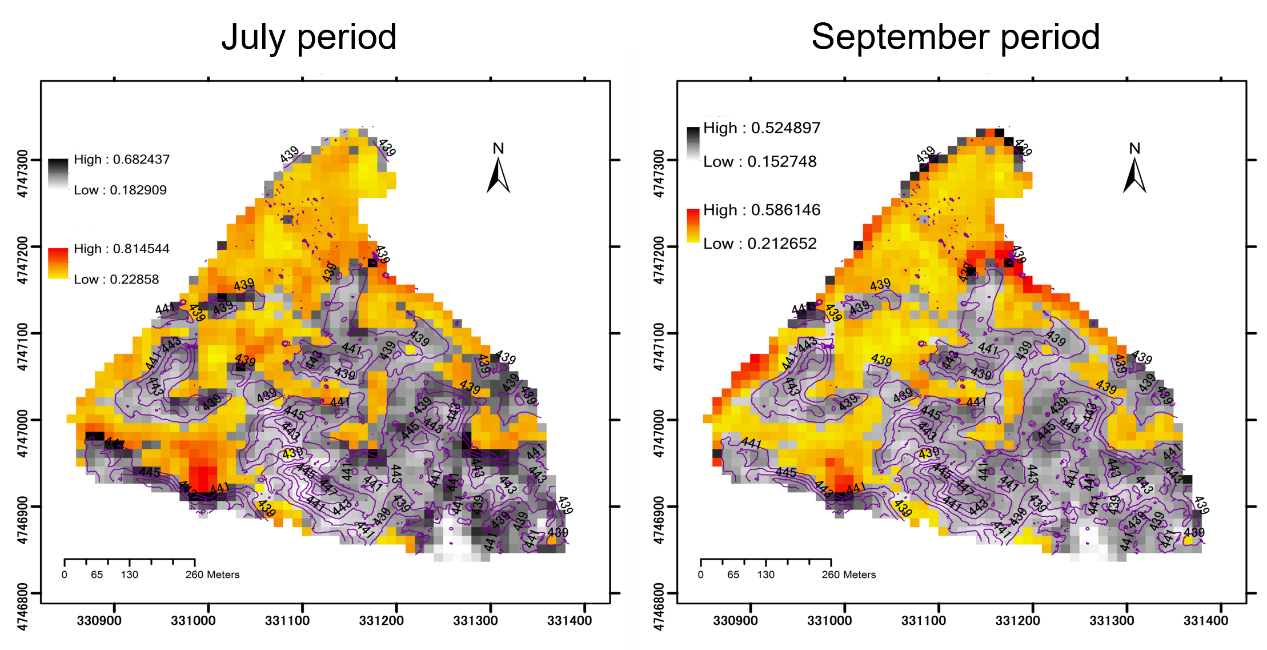


Fig S3. NDVI Distribution in the Study Ranch for July and September Period


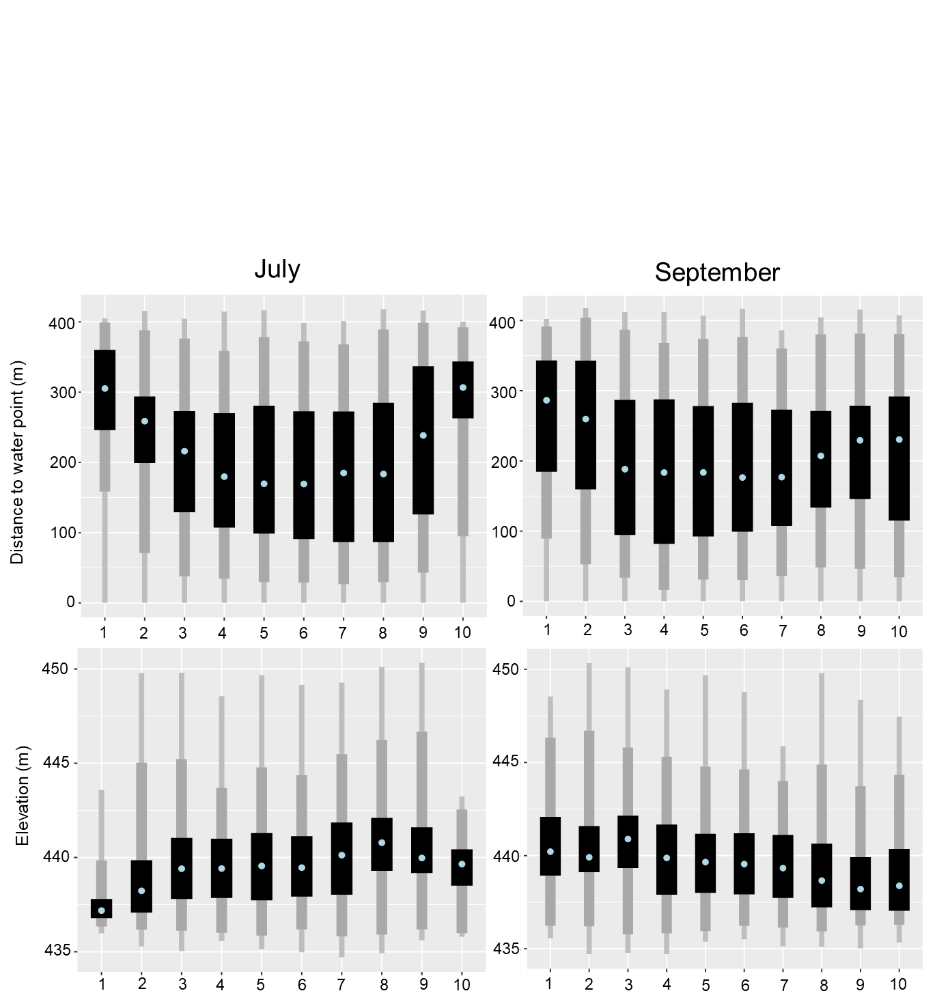


**Fig S4.** Variation of cattle distribution in response to distance to water points and elevation over three-day intervals in July and September.
